# CT radiomics analysis facilitates preoperative risk stratification of central lymph node metastasis in papillary thyroid cancer: a multicenter study

**DOI:** 10.3389/fonc.2025.1681000

**Published:** 2025-11-21

**Authors:** Zeyong Li, Li Zhang, Shuangxin Li, Guishen Jiang, Zhenqi Zhang, Dan Liu, Hongwei Li, Bo Xiao, Jialin Yu

**Affiliations:** 1 Department of Radiology, Bishan Hospital of Chongqing Medical University, Chongqing, China; 2 Department of Radiology, The Affiliated Mianyang Hospital of Chongqing Medical University, Mianyang, China; 3 Department of Radiology, The Second Affiliated Hospital of Army Medical University, Chongqing, China

**Keywords:** papillary thyroid cancer, radiomics, lymph node metastasis, computed tomography, nomogram

## Abstract

**Rationale and objectives:**

To develop a CT-based radiomics model to predict central lymph node metastasis (CLNM) in papillary thyroid cancer (PTC) patients and classify risk.

**Materials and methods:**

218 PTC patients from institution 1 were retrospectively enrolled and randomly assigned to a training set and an internal test set (ratio 7:3). Another 64 patients from institution 2 were assigned to an independent test set. Radiomics features were extracted from the arterial phase CT images of PTC. A radiomics signature (Rad-score) was developed using the least absolute shrinkage and selection operator (LASSO) method. Three models, combined model, clinical model, and Rad-score, were established by logistic regression analysis. These models were comprehensively assessed by the area under the receiver operating characteristic curve (AUC), the calibration curve, and the decision curve analysis (DCA). The improvement in predictive efficacy of the combined nomogram was evaluated using the integrated discrimination improvement index (IDI) and net reclassification improvement index (NRI). The defined threshold of the predicted risk score was set at 0.5, and the stratification effect of the combined nomogram was evaluated by subgroup analysis.

**Results:**

The Rad-score and another three independent predictors (tumor margin, thyroid capsule state and tumor site) were integrated into a combined nomogram. The AUCs of the combined nomogram were 0.848, 0.858, and 0.840 in the training, internal test, and external test sets, respectively, which were greater than those of the clinical model and the Rad-score. The IDI and NRI were greater than 0 indicating better discriminatory accuracy of the combined nomogram than the clinical nomogram and Rad-score. The net benefit of the combined nomogram in the clinical setting was reflected in the DCA. The combined model allows for the effective stratification of patients in diverse risk subgroups.

**Conclusion:**

Combining Rad-score and clinical predictors in an integrated model allow for more accurate prediction of CLNM in PTC patients and enables effective risk stratification.

## 1 Introduction

Papillary thyroid cancer (PTC) is the commonest pathological type of thyroid cancer (1, 2) and generally has a favorable prognosis, but central cervical lymph node metastasis (CLNM) occurs at an incidence of 30%-80% (3, 4), which is strongly associated with tumor recurrence and poor prognosis (5, 6). Therapeutic central lymph node dissection is therefore recommended, but controversy exists over whether prophylactic central lymph node dissection (pCND) should be performed in patients with low-risk PTC (4). pCND facilitates the detection of occult CLNM, provides accurate clinical staging, and reduces the risk of tumor recurrence and reoperation. Relatively, it increases the risk of postoperative complications and may lower the quality of life (7–10). In addition, owing to the high incidence and concealment of CLNM, 11.7% to 63.8% of cases with CLNM are not identified preoperatively (11). For these patients, more aggressive clinical management may be required. Therefore, accurately predicting CLNM preoperatively is crucial.

Ultrasound (US) and computed tomography (CT) are commonly used to assess CLNM among PTC patients. Nevertheless, the effectiveness of US is limited by the presence of the esophagus, trachea, and bones. The sensitivity of US for diagnosing CLNM is poor and can only affect the surgical procedure in 20% of patients (4, 12). CT provides higher sensitivity than US in CLNM evaluation, and the combination of CT and US further improves detection performance, but it is still poor, with a sensitivity of 40%-55% (13–15). In addition, conventional imaging evaluations are susceptible to radiologists’ subjectivity. Moreover, patients with the same clinical features can exhibit different outcomes of CLNM. Thus, it is necessary to further explore methods to accurately predict CLNM and enable risk stratification.

Radiomics, a novel method based on medical imaging, has emerged as a noninvasive approach to assess tumor heterogeneity by quantifying the spatial distribution and gray variation of voxels (16, 17). Recently, it has attracted widespread attention and application in tumor research (18, 19). Several studies have illustrated the availability of CT image-based radiomics to predict CLNM in PTC, but mainly focused on patients with single or micro PTC (20–24), which makes the clinical applicability limited because multifocal cases are quite common in clinical practice. Furthermore, the patients recruited in these studies might exhibit varying degrees of invasive risk, but no studies investigated whether radiomics could stratify CLNM risks in patients with diverse clinical characteristics. Hence, the aim of this research is to develop a preoperative nomogram utilizing CT radiomics and clinical characteristics to predict CLNM among PTC patients and to stratify the risk of CLNM for personalized clinical management.

## 2 Materials and methods

### 2.1 Patients

The research project was approved by the Ethics Committee of Bishan Hospital of Chongqing Medical University (ethical approval code: cqbykyll-20230705-10). The informed consents were waived due to the retrospective nature of this study. Patients with pathologically diagnosed PTC who underwent surgical resection at institution 1 from November 2020 to May 2024 and at institution 2 from August 2021 to December 2023 were retrospectively included in this study. This study adheres to the criteria of METhodological RadiomICs Score (METRICS) (25).

**Figure 1:**
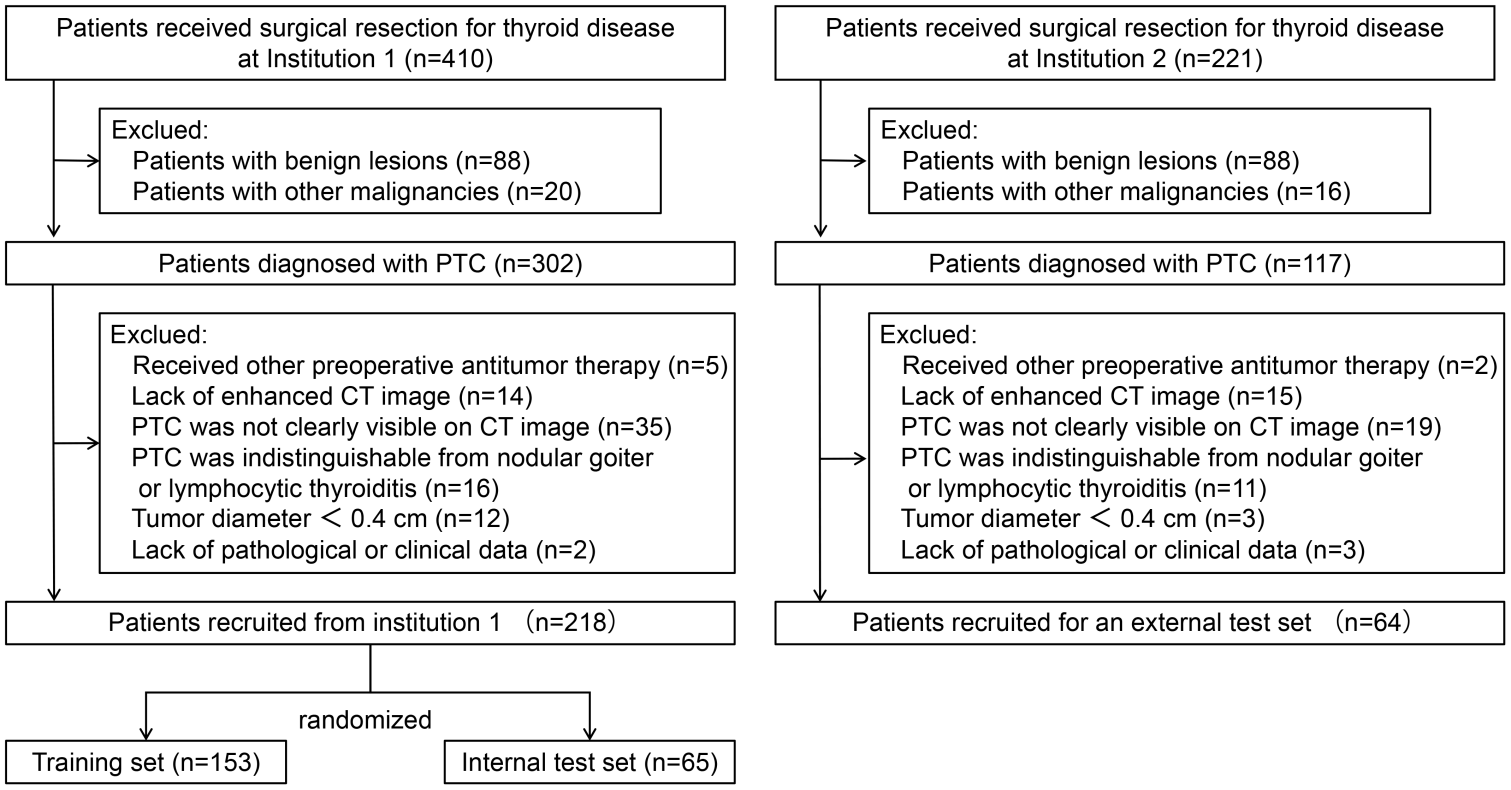
Flowchart of patient recruitment process.

**Figure 2:**
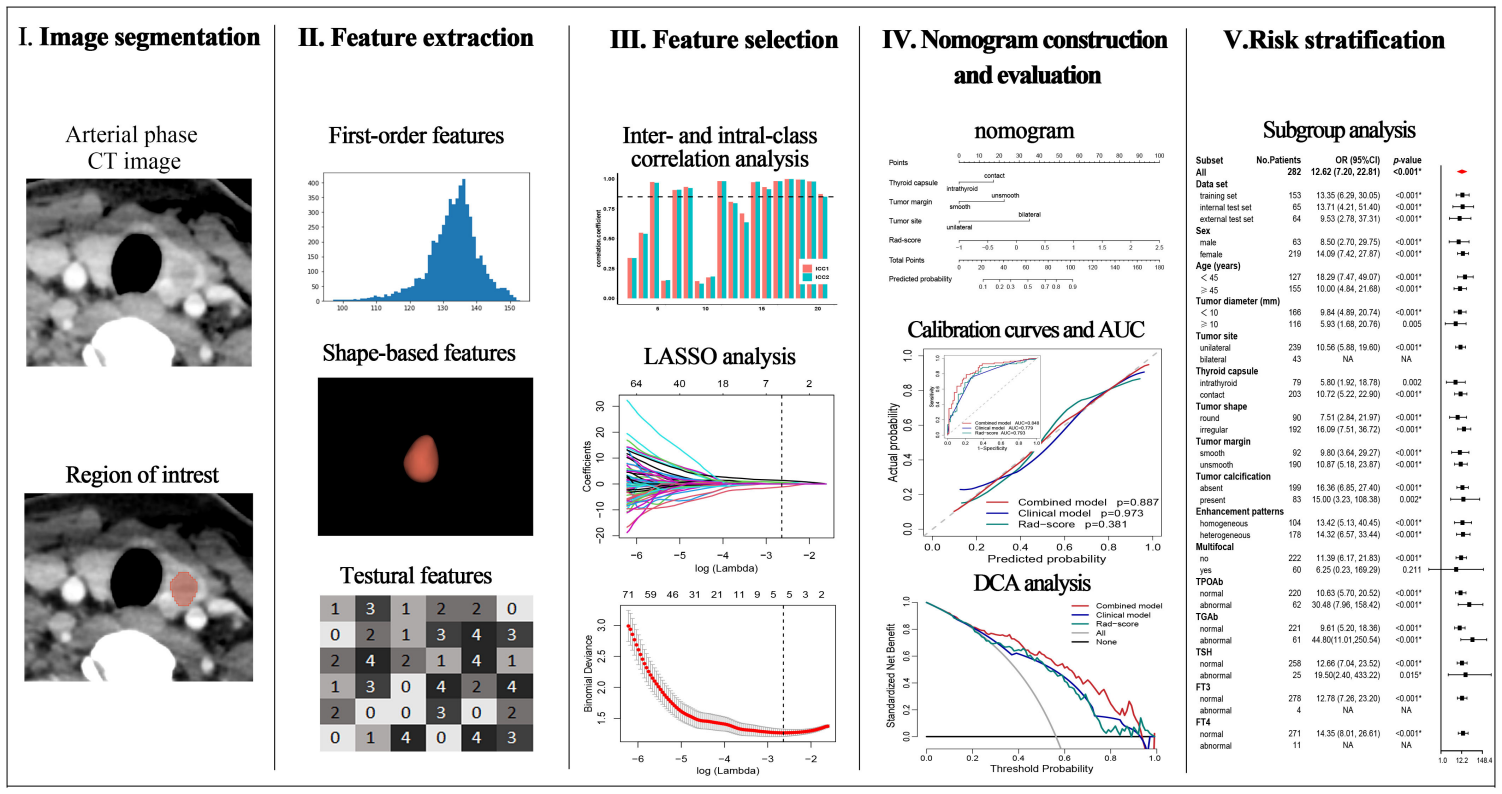
Workflow of the necessary steps in the present study.

The inclusion criteria include (a) patients with PTC confirmed by pathology and no other concomitant malignancy; (b) patients who received thyroidectomy and central neck lymph node dissection. The exclusion criteria include (a) patients who had underwent radiofrequency ablation, radiotherapy, or any other antitumor treatments prior to the current operation; (b) patients without enhanced CT images within 2 weeks prior to surgery; (c) PTC was not clearly visible on CT images; (d) PTC was indistinguishable from nodular goiter or lymphocytic thyroiditis; (e) patients whose maximum tumor diameter was less than 0.4 cm; and (f) patients without complete clinical and pathological information. Ultimately, this study included 282 consecutive PTC patients. Among these, 218 patients recruited from institution 1 were randomized into a training set and an internal test set (ratio 7:3). A total of 64 patients from institution 2 served as an external test set. The patient recruitment process is depicted in **Figure 1** and the study flow is depicted in **Figure 2**.

### 2.2 Image acquisition and evaluation

CT scan procedures were conducted at institution 1 and institution 2 using one of the two 128-slice CT scanners (SOMATOM Definition Flash, SIEMENS; Discovery CT750HD, GE). The technical parameters include 120 kV, 300 mAs or automatic modulation of the tube current, 0.5 s rotation time, 0.6mm or 0.625 mm collimator width, 2.5mm or 5 mm scanning slice thickness, 512×512 matrix, and 0.625mm or 1mm reconstructed slice thickness. After the non-contrast CT scan, 70 mL or 60mL of iohexol (General Electric Pharmaceutical Co., Ltd) and 30 mL of saline were injected sequentially at a rate of 3.0 mL/s. The arterial phase was scanned with 30-second delay, the venous phase with 45-second delay, and the scan was performed from the base of the skull to the subclavian region.

Radiologists 1 (ZL with 7 years of experience in diagnostic radiology) and 2 (LZ with 9 years of experience in diagnostic radiology) independently evaluated the following semantic CT features: tumor site (unilateral or bilateral), tumor diameter (the maximum diameter on axial section), multifocal (yes or no), thyroid capsule (intrathyroid or capsule contact), tumor shape (round and irregular), tumor margin (smooth or unsmooth), tumor calcification (absent or present), and enhancement patterns (homogeneous or heterogeneous). Both radiologists made their assessments independently without knowledge of the patients’ clinical data or follow-up information, and any divergence was resolved through consultation.

### 2.3 Clinical characteristics

The clinical characteristics were as follows: age, sex (female and male), free triiodothyronine (FT3), free thyroxine (FT4), thyroid-stimulating hormone (TSH), thyroglobulin antibody (TGAb), and thyroid peroxidase antibody (TPOAb). The normal standards in institution 1 are as follows: TGAb (<115 IU/mL), TPOAb (< 34 IU/mL), FT3 (3.1–6.8 pmol/L), FT4 (12–22 pmol/L), and TSH (0.27–4.2 µIU/mL). The normal standards in institution 2 are as follows: TGAb (<115 IU/mL), TPOAb (0–5.61 IU/mL), FT3 (2.43–6.01 pmol/L), FT4 (9.01–19.05 pmol/L), and TSH (0.35–4.94 µIU/mL).

### 2.4 Imaging segmentation and radiomics feature extraction

The volume of each primary tumor on arterial phase CT images was manually delineated by Radiologist 1 using 3D Slicer (version 4.9.0; http://www.slicer.org). One month later, radiologists 1 and 2 randomly delineated the tumor of 30 patients to assess the inter- and intra-class consistency of the radiomics features. For patients with multifocal PTC, all visible tumors were delineated.

Radiomics features were extracted via PyRadiomics package (version 2.2.0), including first-order features, shape-based features, texture features, and derived features of the wavelet and Laplacian of Gaussian filters. Before extracting the features, we set normalization to true, the resampling pixel spacing to 1×1×1 mm, the bin width to 25, and the interpolator to sitkNearestNeighbor (18). The radiomics features were standardized by the z score.

### 2.5 Rad-score construction

A Rad-score was constructed based on the training set. Radiomics features with inter- and intra-class correlation coefficients (ICC) greater than 0.85 were screened for the subsequent univariate logistic regression analysis, and the features with a *p*-value less than 0.05 were selected. The logistic regression algorithm of least absolute shrinkage and selection operator (LASSO) was subsequently used to select non-zero coefficient features to construct the Rad-score.

### 2.6 Nomogram development and evaluation

Univariate logistic analyses were performed based on the training set data to select statistically different variables (*p* < 0.05), multivariate logistic analysis was subsequently performed to identify independent risk factors (*p* < 0.05). The variables with variance inflation factor (VIF) greater than 10 were excluded due to multicollinearity (26). The combined nomogram included the Rad-score, clinically and semantic CT independent risk factors, and the clinical model included only clinically and semantic CT independent risk factors.

The models’ performance was evaluated and compared. The predictive efficiency was assessed using the area under the receiver operating characteristic curve (AUC). The incremental predictive value of the combined model was evaluated via integrated discrimination improvement (IDI) and net reclassification improvement (NRI) (27). The concordance between predicted and actual probabilities was assessed by the calibration curve and Hosmer-Lemeshow test (*H-L test*), and the clinical utility was evaluated by the decision curve analysis (DCA) (28). The validation of the combined model was conducted on both the internal and external test sets.

### 2.7 Risk stratification of CLNM

The cutoff value for the predicted risk of the combined model was defined as 0.5. Patients with a predicted risk value > 0.5 were defined as high risk, and those with a value ≤ 0.5 were defined as low risk. Risk stratification analysis was performed based on the entire dataset, which is presented in the form of bar-risk plots and forest plots.

### 2.8 Statistical analysis

R software (version 4.1.3) was used for statistical analysis. The three datasets were compared using analysis of variance (ANOVA) or *Kruskal-Wallis tes*t for continuous variables and *Chi-square test* or *Fisher’s exact test* for categorical variables. LASSO analysis was conducted via the ‘glmnet’ package. Logistic regression analyses were performed with the ‘MASS’ package. The calibration curves were constructed via the ‘rms’ package. the AUC calculation and DeLong-test were performed using the ‘pROC’ package. The IDI and NRI were computed with the ‘PredictABEL’ package. The H-L test was performed with the ‘ResourceSelection’ package. The DCA was executed with the ‘rmda’ package. A two-tailed *p*-value less than 0.05 indicates statistical significance.

## 3 Results

### 3.1 Demographics characteristics

Ultimately, 282 patients were included in this study, including 153 in the training set (male: 36, female: 117, age range: 22–72), 65 in the internal test set (male: 12, female: 53, age range: 28–69), and 64 in the external test set (male: 15, female: 49, age range: 22–74). The clinical baseline data are presented in **Table 1**. The CLNM rates were 56.2% (86/153), 64.4% (42/65), and 68.8% (44/64) in the training, internal test, and external test sets (*p* = 0.178), respectively.

**Table 1:** Baseline characteristics of the training, internal test, and external test sets.

Parameter	Training set (n=153)	Internal test set (n=65)	External test set (n=64)	*P*-value
CLNM				0.178
positive	86 (56.2)	42 (64.6)	44 (68.8)	
negative	67 (43.8)	23 (35.4)	20 (31.2)	
Age (years)				0.033
<45	60 (39.2)	38 (58.5)	29 (45.3)	
≥45	93 (60.8)	27 (41.5)	35 (54.7)	
Sex				0.693
female	117 (76.5)	53 (81.5)	49 (76.6)	
male	36 (23.5)	12 (18.5)	15 (23.4)	
Tumor diameter (mm)				0.001
<10	101 (66.0)	40 (61.5)	25 (39.1)	
≥10	52 (34.0)	25 (38.5)	39 (60.9)	
Tumor site				0.034
unilateral	136 (88.9)	55 (84.6)	48 (75.0)	
bilateral	17 (11.1)	10 (15.4)	16 (25.0)	
Thyroid capsule				0.514
intrathyroidal	47 (30.7)	17 (26.2)	15 (23.4)	
contact	106 (69.3)	48 (73.8)	49 (76.6)	
Tumor shape				0.889
round	47 (30.7)	22 (33.8)	21 (32.8)	
irregular	106 (69.3)	43 (66.2)	43 (67.2)	
Tumor margin				0.254
smooth	44 (28.8)	26 (40.0)	22 (34.4)	
unsmooth	109 (71.2)	39 (60.0)	42 (65.6)	
Tumor calcification				0.144
absent	111 (72.5)	49 (75.4)	39 (60.9)	
present	42 (27.5)	16 (24.6)	25 (39.1)	
Multifocal				0.011
no	130 (85.0)	49 (75.4)	43 (67.2)	
yes	23 (15.0)	16 (24.6)	21 (32.8)	
Enhancement patterns				0.184
homogeneous	63 (41.2)	23 (35.4)	18 (28.1)	
heterogeneous	90 (58.8)	42 (64.6)	46 (71.9)	
TPOAb				0.777
normal	120 (78.4)	52 (80.0)	48 (75.0)	
abnormal	33 (21.6)	13 (20.0)	16 (25.0)	
TGAb				0.601
normal	122 (79.7)	48 (73.8)	51 (79.7)	
abnormal	31 (20.3)	17 (26.2)	13 (20.3)	
FT3				0.535
normal	150 (98.0)	64 (98.5)	64 (100.0)	
abnormal	3 (2.0)	1 (1.5)	0 (0.0)	
FT4				0.909
normal	147 (96.1)	62 (95.4)	62 (96.9)	
abnormal	6 (3.9)	3 (4.6)	2 (3.1)	
TSH				0.660
normal	142 (92.8)	58 (89.2)	58 (90.6)	
abnormal	11 (7.2)	7 (10.8)	6 (9.4)	
Rad-score	0.34 ± 0.63	0.51 ± 0.64	0.68 ± 0.70	0.001

Normally distributed variables are presented as the mean ± standard deviation, and categorical variables are presented as n (%).

CLNM, central cervical lymph node metastasis; TPOAb, thyroid peroxidase antibody; TGAb, anti-thyroglobulin antibodies; FT3, free triiodothyronine; FT4, free tetraiodothyronine; TSH, thyroid stimulating hormone; Rad-score, radiomics signature.

The normal standards in institution 1 were as follows: TGAb (<115 IU/mL), TPOAb (< 34 IU/mL), FT3 (3.1–6.8 pmol/L), FT4 (12–22 pmol/L), and TSH (0.27–4.2 µIU/mL). The normal standards in institution 2 were as follows: TGAb (<115 IU/mL), TPOAb (0–5.61 IU/mL), FT3 (2.43–6.01 pmol/L), FT4 (9.01–19.05 pmol/L), and TSH (0.35–4.94 µIU/mL).

### 3.2 Rad-score construction

**Figure 3:**
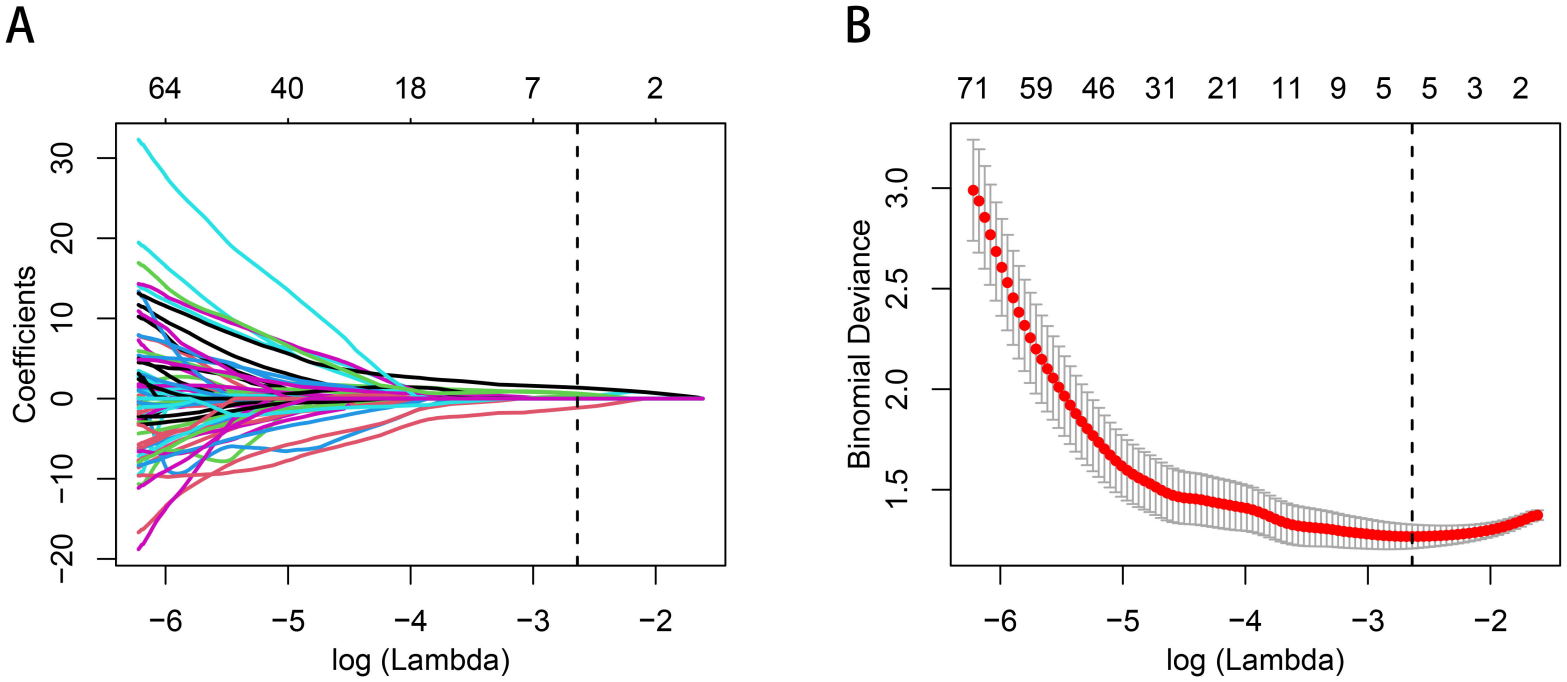
Radiomics features selection by using the logistic regression algorithm of LASSO. **(A)** Coefficients convergence plot of the radiomics features. **(B)** Penalty parameter selection by using 10-fold cross-validation via criteria of minimum bias variance. The dotted vertical line indicates the optimal Lambda value of 0.07145 (ln (Lambda)= -2.63875) resulting in five features with nonzero coefficients.

A total of 1070 radiomics features were calculated, of which 771 features with ICC > 0.85. 181 features with a *p*-value less than 0.05 were then selected by univariate analysis, and 5 features were ultimately screened via LASSO to generate the Rad-score (**Figure 3**). The arithmetic formula of the Rad-score is presented in **Table 2**.

**Table 2:** Radiomics features selected for Rad-score construction.

Radiomics feature name	Coefficient
(Intercept)	-0.47752046
Wavelet.HHH_glcm_MaximumProbability	-1.146981297
Wavelet.HHH_glrlm_RunEntropy	0.288989842
Log.sigma.2.0.mm.3D_glcm_MCC	0.588084624
Wavelet.LLL_glcm_Correlation	0.641078365
Wavelet.HLL_glszm_ZoneEntropy	1.364064898

Rad-score=-0.47752046.

-1.146981297×Wavelet.HHH_glcm_MaximumProbability.

+0.288989842×Wavelet.HHH_glrlm_RunEntropy.

+0.588084624×Log.sigma.2.0.mm.3D_glcm_MCC.

+0.641078365×Wavelet.LLL_glcm_Correlation.

+1.364064898×Wavelet.HLL_glszm_ZoneEntropy.

### 3.3 Nomogram construction

**Figure 4:**
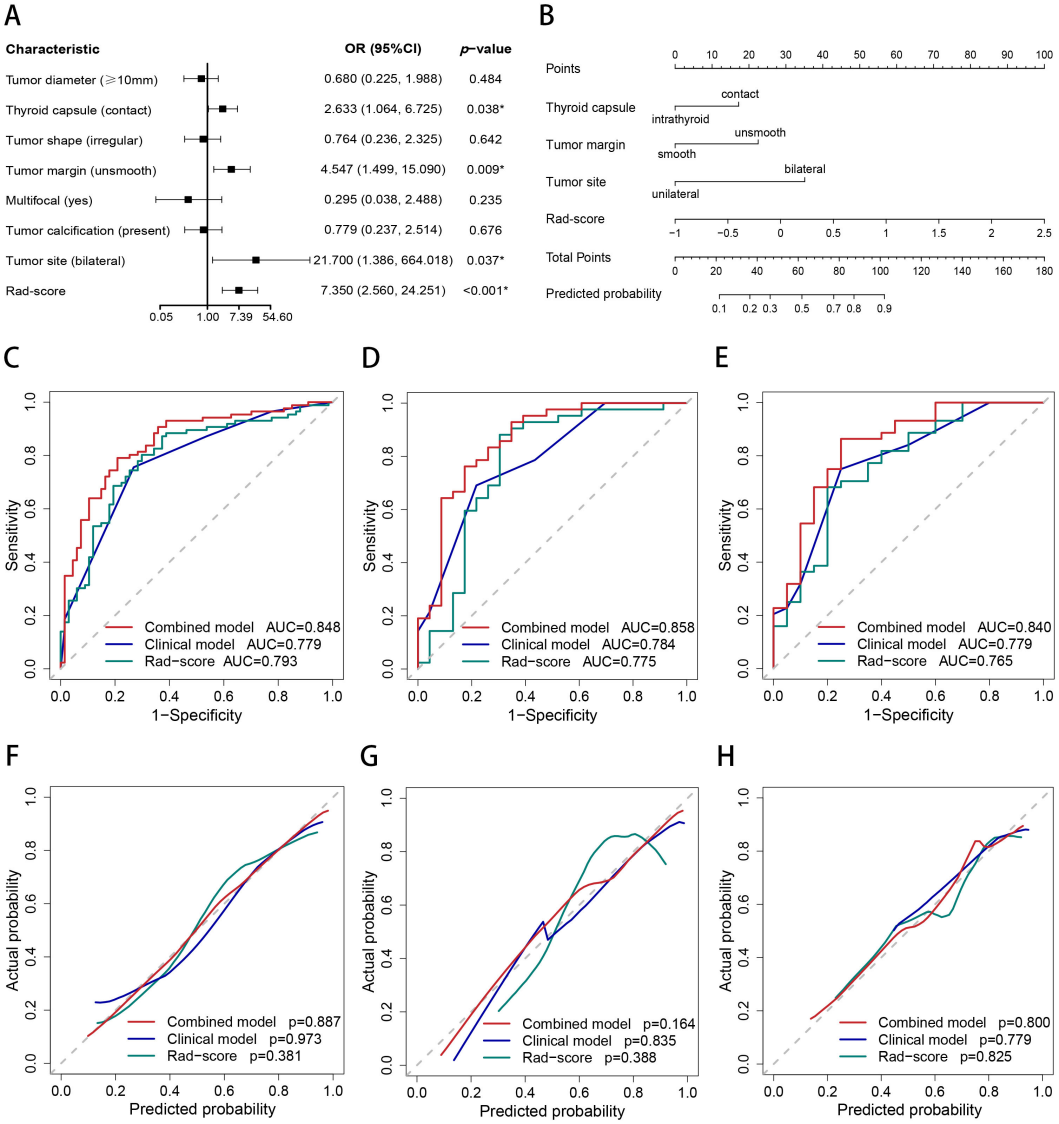
Nomogram, AUC, and calibration curves. **(A)** The forest plot for multivariate logistic regression analysis. **(B)** The nomogram of the combined model. The AUC of the combined model, clinical model, and Rad-score in the training **(C)**, internal test **(D)**, and external test **(E)** sets. The calibration curves of the combined model, clinical model, and Rad-score in the training **(F)**, internal test **(G)**, and external test **(H)** sets.

The outcomes of the logistic regression analyses, both univariate and multivariate, are presented in **Table 3**. The variables that were statistically significant in the univariate analysis include the Rad-score, tumor diameter, tumor site, thyroid capsule, tumor shape, tumor margin, multifocal and calcification. The independent predictors identified by multivariate analysis (*p* < 0.05) include the Rad-score, tumor margin, tumor site and thyroid capsule state (range of VIF: 1.00-1.05). The Rad-score, tumor margin, tumor site and thyroid capsule were integrated into a combined nomogram (**Figures 4A, B**). The tumor margin, tumor site and thyroid capsule were integrated into a clinical model.

**Table 3:** Univariate and multivariate logistic regression analyses.

Variable	Univariate analysis		Multivariate analysis	
	OR (95%CI)	*P* value	OR (95%CI)	*P*-value
Age (≥45)	0.78 (0.40-1.49)	0.448		
Sex (male)	1.30 (0.61-2.84)	0.498		
Tumor diameter (≥10mm)	3.99 (1.92-8.76)	<0.001*	0.68 (0.23-1.99)	0.484
Tumor site (bilateral)	15.09(2.95-276.07)	0.009*	21.70(1.39-664.01)	0.037*
Multifocal (yes)	4.47 (1.58-16.05)	0.010*	0.29 (0.04-2.49)	0.235
Thyroid capsule (contact)	4.99 (2.41-10.80)	<0.001*	2.63 (1.06-6.73)	0.038*
Tumor shape (irregular)	2.53 (1.26-5.18)	0.010*	0.76 (0.24-2.32)	0.642
Tumor margin (unsmooth)	4.84 (2.30-10.64)	<0.001*	4.55 (1.50-15.09)	0.009*
Tumor calcification (present)	3.38 (1.56-7.85)	0.003*	0.78 (0.24-2.51)	0.676
Enhancement patterns (heterogeneous)	0.82 (0.36-1.84)	0.425		
TPOAb (abnormal)	0.67 (0.31-1.46)	0.314		
TGAb (abnormal)	0.67 (0.30-1.49)	0.327		
FT3	1.01 (0.79-1.35)	0.912		
FT4	0.98 (0.88-1.08)	0.651		
TSH	1.08 (0.88-1.38)	0.493		
Rad-score	7.79 (3.80-17.82)	<0.001*	7.35 (2.56-24.25)	<0.001*

TPOAb: thyroid peroxidase antibody, TGAb: anti-thyroglobulin antibodies, FT3: free triiodothyronine, FT4: free tetraiodothyronine, TSH: thyroid stimulating hormone, Rad-score: radiomics signature.

*p < 0.05.

### 3.4 Performance of the models

**Figure 5:**
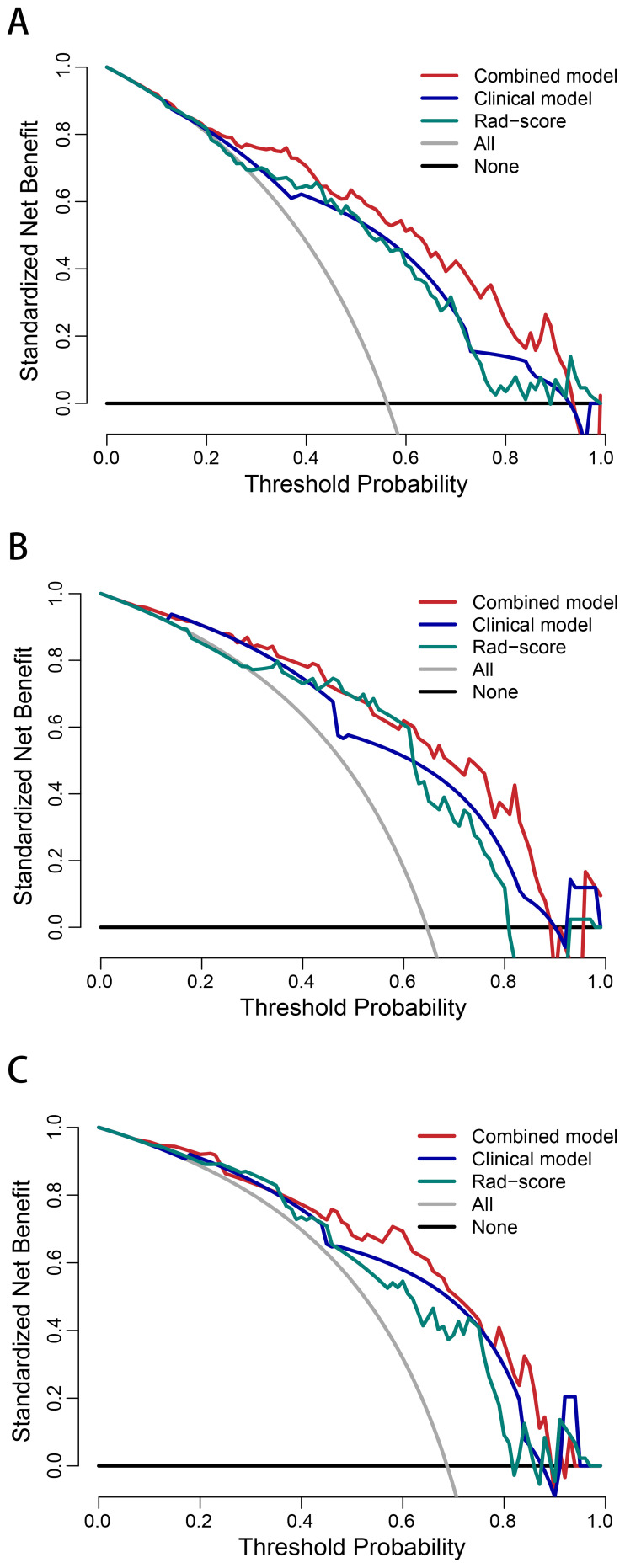
Decision curve analysis of the combined model, clinical model, and Rad-score in the training **(A)**, internal test **(B)**, and external test **(C)** sets.

The AUCs of the combined model were 0.848 (95% confidence interval [CI]: 0.786–0.911), 0.858 (95% CI: 0.755–0.962), and 0.840 (95% CI: 0.728–0.952) in the training, internal test, and external test sets, respectively, which were greater than those of the clinical model and the Rad-score (**Table 4** and **Figures 4C–E**). The IDI and NRI were greater than 0 indicating that the classification accuracy of the combined model was improved by combining clinical predictors and Rad-score (**Table 5**). The calibration curves revealed that the predicted probabilities of the combined model for CLNM were well consistent with the actual probabilities (*H-L test >*0.05) (**Figures 4F–H**). The DCA results indicated that the combined model had favorable clinical utility (**Figure 5**).

**Table 4:** The AUC of models in the training set, internal test set, and external test set.

	AUC (95% CI)	Sensitivity (95% CI)	Specifcity (95% CI)	*P*-value (DeLong-test)
Training set				
Combined model	0.848 (0.786-0.911)	0.791 (0.705-0.877)	0.791 (0.694-0.888)	reference
Clinical model	0.779 (0.709-0.849)	0.756 (0.665-0.847)	0.731 (0.625-0.837)	0.005*
Rad-score	0.793 (0.720-0.866)	0.802 (0.718-0.886)	0.701 (0.592-0.811)	0.019*
Internal test set				
Combined model	0.858 (0.755-0.962)	0.762 (0.633-0.891)	0.826 (0.671-0.981)	reference
Clinical model	0.784 (0.670-0.898)	0.690 (0.551-0.830)	0.783 (0.614-0.951)	0.117
Rad-score	0.775 (0.637-0.914)	0.881 (0.783-0.979)	0.696 (0.508-0.884)	0.118
External test set				
Combined model	0.840 (0.728-0.952)	0.864 (0.762-0.965)	0.750 (0.560-0.940)	reference
Clinical model	0.779 (0.658-0.901)	0.750 (0.622-0.878)	0.750 (0.560-0.940)	0.132
Rad-score	0.765 (0.632-0.898)	0.682 (0.544-0.819)	0.800 (0.625-0.975)	0.089

AUC: area under the receiver operating characteristic curves, CI: confidence interval, Rad-score: radiomics signature.

*p < 0.05.

**Table 5:** The NRI and IDI of Combined model compared with Clinical model and Rad-score.

Model comparison	NRI (95% CI)	*P*-value	IDI (95% CI)	*P*-value
Training set				
Combined model VS Clinical model	0.616 (0.312-0.920)	<0.001*	0.102 (0.055-0.149)	<0.001*
Combined model VS Rad-score	0.752 (0.456-1.047)	<0.001*	0.113 (0.064-0.161)	<0.001*
Internal test set				
Combined model VS Clinical model	0.694 (0.214-1.173)	0.005*	0.092 (-0.001-0.185)	0.050
Combined model VS Rad-score	0.899 (0.457-1.340)	<0.001*	0.110 (0.033-0.187)	0.005*
External test set				
Combined model VS Clinical model	0.527 (0.052-1.002)	0.030*	0.084 (-0.011-0.180)	0.084
Combined model VS Rad-score	0.664 (0.163-1.164)	0.009*	0.107 (0.027-0.187)	0.009*

NRI: net reclassification improvement, IDI: integrated discrimination improvement index, CI: confidence interval.

*p < 0.05.

### 3.5 Risk stratification

**Figure 6:**
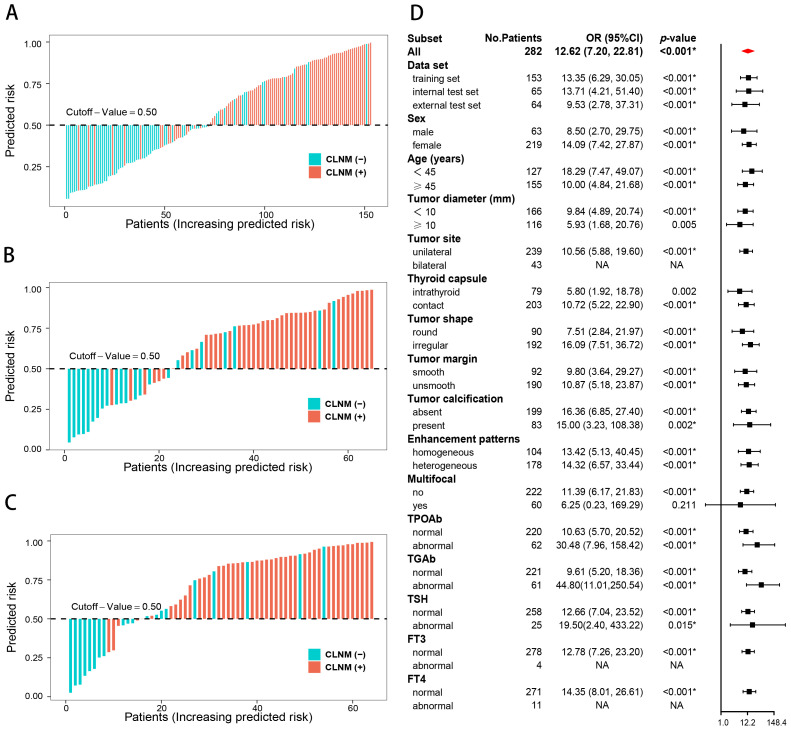
Risk stratification of the combined model. Risk bar charts for the training **(A)**, internal test **(B)**, and external test **(C)** sets. The forest plot of the subgroup analysis for the risk stratification **(D)**.

The CLNM rates were significantly different between the high- and low-risk groups of PTC patients in the training (*p* < 0.001; odds ratio [OR] = 13.35, 95% CI: 6.29–30.05), internal test (*p* < 0.001; OR = 13.71, 95% CI: 4.21–51.40), and external test (*p* < 0.001; OR = 9.53, 95% CI: 2.78–37.31) datasets. The subgroup analyses demonstrated that the combined model enables subgroup risk stratification of CLNM among PTC patients with different clinical characteristics. For instance, regardless of whether the tumor diameter was less than 1 cm (*p* < 0.001; OR = 9.84, 95% CI: 4.89–20.74) or greater than 1 cm (*p* = 0.005; OR = 5.93, 95% CI:1.68–20.76), or whether the PTC was intrathyroid (*p* = 0.002; OR = 5.80, 95% CI: 1.92–18.78) or thyroid capsule contact (*p* < 0.001; OR = 10.72, 95% CI:5.22–22.90), the combined model could effectively perform stratification (**Figure 6**).

## 4 Discussion

In this study, a Rad-score based on arterial-phase CT images was recognized as a valid biomarker of CLNM in PTC and served to improve the predictive efficacy of the combined model. The combined model enables subgroup risk stratification of CLNM among PTC patients with different clinical characteristics.

For the decision-making process in clinical practice, accurate assessment of CLNM is crucial (5, 6). Consistent with several previous studies (20–22), the Rad-score based on CT images serves as an independent predictor for CLNM, and helps improve the predictive efficacy of the nomogram. The reason for this is that radiomics can quantify tumor heterogeneity by characterizing the spatial distribution and gray variation of voxels, which strongly correlates with the biological aggressiveness of tumors (16, 17, 29, 30). In our study, five radiomics features were selected for Rad-score construction. These features characterize the randomness and uncertainty in the distribution of zone sizes, run lengths, and gray levels of voxels in the CT images, which reflects the spatial heterogeneity of tumors in multiple dimensions. And the close connection between the radiomics features and the biological characteristics of PTC has been revealed by previous studies at the genetic and molecular levels (21, 23, 31).

Although previous studies have suggested that radiomics analysis based on CT images is an effective method for predicting CLNM in PTC patients, relevant studies are still limited (20–24). Peng et al. (20) reported the usefulness of CT radiomics in predicting CLNM for cN0 PTC patients, but the study was a single-center research with a small sample size and did not include clinical features. Several high-quality studies have further explored and validated the good predictive performance of the radiomics, while these studies mainly focused on patients with single or micro PTC and the role of the radiomics for risk stratification in subgroups of diverse clinical characteristics remains unclear (21–24). In this study, we extended the use of radiomics to patients with multiple or bilateral lesions. The combined model based on Rad-score achieved good efficacy for CLNM prediction and outperforms clinical model and Rad-score. Furthermore, subgroup analysis in this study indicated that the combined model could effectively stratify CLNM risk among nearly all clinical characteristics. However, we noted that the risk stratification for multifocal and bilateral lesions lost its statistical efficacy. This is because most patients with multifocal or bilateral PTC in this study exhibited CLNM, and nearly all of them achieved accurate prediction and classification. This may also be related to selection bias or relatively limited sample size, ultimately resulting in a lack of sufficient low-risk control patients with multifocal or bilateral lesions. But this does not affect the model’s generalizability for both unifocal and multifocal PTC, as its good predictive performance was validated in an external validation group. While it is still necessary to conduct further research on multifocal PTC as an independent topic.

Furthermore, the tumor margin, thyroid capsule state and tumor site were independent predictors of CLNM in this study. Consistent with previous studies (22, 32, 33), unsmooth margin, thyroid capsule contact and bilateral PTC suggested a high risk of CLNM. An unsmooth margin reflects the more aggressive of malignant cells (32). Similarly, bilateral PTC is more aggressive than unilateral PTC, exhibiting a higher rate of lymph node metastasis and a worse prognosis (33). The sign of thyroid capsule contact is closely associated with extrathyroidal extension in PTC (34, 35), while extrathyroidal extension is a well-established prognostic biomarker for CLNM (11, 36). It is worth noting that tumor diameter, multifocal, tumor shape, and calcification were independent predictors of CLNM in previous studies (21, 23, 32, 37, 38), but these variables lost statistical significance in the multivariate analysis of this study. This is attribute to the difference of cases composition and the interaction among variables. For example, although tumor diameter greater than 1cm is generally considered as a risk factor for CLNM, a considerable portion of patients with tumor diameter less than 1cm still develop CLNM in clinical practice, and the incidence was reported to be 20.7%~62% (21). In our training dataset, the CLNM rate was 45.5% (46/101) in PTC patients with tumor diameter less than 1cm. Thus, the statistical significance of these variables were masked. And precisely the difference in patients’ characteristics and the interaction among variables further illustrate the importance and necessity of effective risk stratification for PTC patients.

There are several limitations in this study. First, this is a retrospective study, which may be associated with greater patient selection bias. Second, even though this is a multicenter study, the sample size is still relatively limited. Third, the pathological and molecular biological basis of radiomics features is not addressed in this paper. Therefore, further validation and improvement through large sample, prospective studies is necessary. It is also necessary to further reveal the molecular biology and pathological nature of radiomics.

## 5 Conclusion

Rad-score is a valid biomarker of CLNM in PTC patients and contributes to improving the predictive efficiency of the combined model. The combined model enables subgroup risk stratification of CLNM. This allows for personalized evaluation of CLNM risk preoperatively, thus facilitating personalized clinical management for PTC patients.

## Data Availability

The raw data supporting the conclusions of this article will be made available by the authors, without undue reservation.
